# Predictive Factors for the Effect of Treatment by Noninvasive Ventilation in Patients with Respiratory Failure as a Result of Acute Exacerbation of the Chronic Obstructive Pulmonary Disease

**DOI:** 10.3889/oamjms.2015.115

**Published:** 2015-11-11

**Authors:** Sava Pejkovska, Biserka Jovkovska Kaeva, Zlatica Goseva, Zoran Arsovski, Jelena Jovanovska Janeva, Sead Zeynel

**Affiliations:** *University Clinic of Pulmonology and Allergology, Faculty of Medicine, Ss Cyril and Methodius University of Skopje, Skopje, Republic of Macedonia*

**Keywords:** COPD, noninvasive ventilation, respiratory failure, predictive factors, COPD exacerbation

## Abstract

**BACKGROUND:**

Noninvasive mechanical ventilation (NIV) applies ventilator support through the patient’s upper airway using a mask.

**AIM:**

The aim of the study is to define factors that will point out an increased risk of NIV failure in patients with exacerbation of Chronic Obstructive Pulmonary Disease (COPD).

**PATIENTS AND METHODS:**

Patients over the age of 40, treated with NIV, were prospectively recruited. After data processing, the patients were divided into two groups: 1) successful NIV treatment group; 2) failed NIV treatment group.

**RESULTS:**

On admission arterial pH and Glasgow coma scale (GCS) levels were lower (pH: p < 0.05, GCS: p < 0.05), and Acute Physiology and Chronic Health Evaluation II (APACHE) score and PaCO_2_ were higher (p < 0.05) in the NIV failure group. Arterial pH was lower (p < 0.05) and PaCO_2_ and respiratory rate were higher (p < 0.05) after 1h, and arterial pH was lower (p < 0.05) and PaCO_2_ (p < 0.05), respiratory and heart rate were higher (p < 0.05) after 4h in the NIV failure group.

**CONCLUSION:**

Measurement and monitoring of certain parameters may be of value in terms of predicting the effectiveness of NIV treatment.

## Introduction

Chronic Obstructive Pulmonary disease (COPD) is one of the commonest diseases in the world. It is an increasing international health problem with a projected third leading cause of mortality within the adult population [1]. Chronic obstructive pulmonary disease is a respiratory disorder largely caused by smoking, and is characterized by progressive, partially reversible airway obstruction and lung hyperinflation, systemic manifestations, and increasing frequency and severity of exacerbations. An acute exacerbation is defined as a sustained worsening of dyspnea, cough or sputum production leading to an increase in the use of maintenance medications and/or supplementation with additional medications [2]. The management of the acute exacerbations of COPD accounts for a large proportion of the health care costs because of the need for prolonged hospitalizations and increased rate of mortality [3]. An important event in the course of the disease is the shortening of the inspiratory time, leading to a decrease of the Total Lung Capacity (TLC) and increase of the respiratory rate. The management which aims to increase the TLC with subsequent increase in the alveolar ventilation, as well as the decrease of the respiratory rate, is expected to reverse the impaired respiratory physiology [4].

The conventional management includes continuous oxygen therapy and treatment of the underlying cause of the exacerbation in order to decrease the airway resistance by using bronchodilators, anti-inflammatory drugs, oxygen and antibiotics.

The physiological benefit from the oxygen supplementation is represented by the decrease in the hypoxic pulmonary vasoconstriction, decrease of the pulmonary arterial pressure, thus preventing the right heart strain and cardiac ischemia.

The oxygen supplementation may worsen the hypercapnia, and according to some authors in terms of this occurrence, the dominant role has the increased ‘dead space’, therefore leading to the ventilation/perfusion mismatch. Unfortunately, there is limited possibility to reverse the worsened condition by standard management, whereas it was not long ago when the sole alternative has been the invasive mechanical ventilation associated with a large number of complications and side effects.

In the recent two decades there is particular interest towards the usage of Noninvasive ventilation (NIV) in the management of acute exacerbations of COPD (AECOPD) that require inpatient treatment. NIV applies ventilator support through the patient’s upper airway using a mask, without endotracheal intubation and represents alternative to the management with invasive ventilation in patients with hypercapnic respiratory failure (type II).

NIV is an intermittent model of ventilatory support which lasts from few hours per day (6-12 h), allowing the patient to be fed, and he/she is able to speak. Moreover, the usage of NIV correlates with decrease of the complication rate, especially hospital-acquired pneumonia (in comparison with the intubated patients) [12], as well as decrease in the rate of intubations [7, 9, 11] and mortality [7, 8, 11].

NIV is not always successful. The failure rate is estimated between 5-40% [5]. Treatment failure is commonly due to the fact that the disease is in its terminal phase. Nevertheless, the clinician’s experience and expertise are associated with higher rate of success [6]. However, in case of increased need for ventilatory support, the tolerance of the mask becomes limited, which may be the reason for treatment failure. According to some authors, the NIV tolerance is the sole prognostic factor [13].

The aim of the study is to define the clinical and laboratory indicators that will point out an increased risk of worsening the condition and treatment failure, during NIV treatment of patients with acute respiratory failure due to AECOPD. That will enable urgent implementation of adequate medical actions leading to decreasing mortality. The continuation of NIV treatment in patients with no evident success may delay the invasive mechanical ventilation that will lead to increased mortality.

## Patients and methods

Between October, 2013 and March, 2014, 58 patients, over the age of 40, hospitalized because of acute exacerbation of COPD and treated with NIV were prospectively recruited from University Clinic of Pulmonology and Allergy- Skopje. The COPD diagnosis has been established according to the current guidelines [14].

- **Inclusion criteria:** severe dyspnea (mMRC scale = 3 or 4); respiratory rate > 25/min; hypoxemia (PaO_2_ < 7.3 kPa); hypercapnia (PaO_2_ > 6.1 kPa); respiratory acidosis (pH < 7.35).

- **Exclusion criteria:** indication for emergent endotracheal intubation (respiratory or cardiac arrest); Glasgow coma scale < 8; hemodynamic instability (hypovolemic shock; acute cardiac ischemia; arrhythmias), impaired consciousness; confusion, agitation, face or chest trauma, recent surgical intervention of the face, upper airway and upper gastrointestinal tract; fixed obstruction of the upper airway, vomiting; APACHE II > 29; pregnancy.

### Protocol for performing NIV

Firstly, patients were treated with medications and oxygen therapy using nasal cannula no more than one hour. Patients that didn’t improve in terms of acid-base status were initiated with NIV treatment.

NIV was carried out through a ventilatory support system (BiPAP ST/D, Respironiks). Patient was positioned in a semi-upright sitting position (45° degrees) in order to minimize the risk of pulmonary aspiration. Oronasal or face mask has been used. Expiratory pressure has been at its minimum (4 cmH_2_O), while the inspiratory pressure was at 10 cmH_2_O. The inspiratory pressure had been increased for 2 cmH_2_O in all patients, until the patient showed signs of discomfort (dyspnea) or increased air leak out of the face mask occurred, or until the pressure of 20 cmH_2_O was reached. Similarly, the expiratory pressure had been raised, that is, until the appearance of discomfort or the pressure of 7 cmH_2_O was reached. The oxygen had been delivered until the saturation was 90%. The NIV treatment has been carried out during three days or more, depending of the clinical indications.

The treatment failure has been defined as death or necessity of invasive mechanical ventilation. Its manifestations were worsening of the clinical picture that is, worsening of: 1) pH < 0.04 and PaCO_2_ > 0.08; 2) coma or seizure disorders; 3) haemodynamic instability (heart rate < 50 bpm/and or systolic blood pressure < 70 mmHg; and 4) agitation and inability to tolerate the mask.

The treatment success has been defined as improvement in the acid-base, as well as clinical status and reversal of the condition at the level present before the exacerbation. Thus, oxygen saturation was expected to be measured > 85% without nasal cannula (i.e. > 90% with nasal cannula and oxygen 1-2 L/min); pH > 7.35; RR < 25/min. without the engagement of the accessory respiratory musculature. The commencement of weaning from the NIV was performed with greater pauses during the day or the NIV was used only during the night.

The following data were analyzed: demo exacerbation; Glasgow Coma Scale (GCS); Acute Physiology and Chronic Health Evaluation (APACHE) II score; Respiratory Rate (RR); pH; partial pressure of oxygen (PaO_2_); partial pressure of carbon dioxide (PaCO_2_). Arterialized blood sample was drawn from earlobe and acid-base status was evaluated at the beginning of NIV treatment, 1 hour and 4 hours after, and at the end of the treatment.

### Statistical analysis

The results were statistically analysed according to the Difference test. The results were expressed as mean ± standard deviation. The significances values were taken p < 0.05.

## Results

Out of 85 patients hospitalized because of respiratory failure due to AECOPD, 58 patients have met the inclusion criteria. The treatment success was recorded in 40 patients (68.9%), whereas treatment failure was a case in 18 patients out of the total number. There was no correlation between the patients’ demographics and the treatment’s failure. Treatment complications where observed in two patients with ulcerations on the bridge of the nose and conjunctivitis in one patient. These complications where overcame by changing the mask.

**Table 1:** Demographic and laboratory characteristics at admission

Total patients randomized	58

Age (main ± SD)	65.6 ± 9

Sex (male) [n(%)]	48 (82.7%)

Cause of exacerbation [No (%)]	

Upper respiratory tract infections including bronchitis	38 (65.5%)

Pneumonia	7 (12%)

Other	8 (13.7)

Co-morbidities [n(%)]	

CVDs*	36 (62%)

Diabetes Mellitus	7 (12.0%)

Obesity	19 (32.7)

Hypertension	28 (48.2)

Cachexia	2 (3.4%)

Respiratory rate (main ± SD)	32.5 ± 5

Heart rate (bpm) (main ± SD)	112 ± 8

pH (main ± SD)	7.25 ± 0.08

PaO_2_ kPa (main ± SD)	6.2 ± 1.0

PaCO_2_ kPa (main QUOTE ± SD)	9.7 ± 2.4

APACHE** II (main ± SD)	18.3 ± 6.1

GSC*** (main ± SD)	12.2 ± 2.8

APACHE

** Acute Physiology and Chronic Health Evaluation

CVD

* cardiovascular diseases

GCS

*** Glasgow coma scale.

Upon data processing, patients were divided into two groups: Group 1 – successful NIV treatment and Group 2 – failed NIV treatment.

**Table 2:** Comparison of the clinical and laboratory characteristics between both groups at the time of admission, Group 1 and Group 2

Parameters	Group1 successful NIV (n=40)	Group 2 failed NIV (n=18)	P-value
Respiratory rate	32.3 ± 3.2	34.5 ± 3.0	NS
Heart rate	110 ± 6.1	115 ± 5.2	NS
pH	7.30 ± 0.03	7.23 ± 0.06	p < 0.05
PaO_2_ kPa	6.3 ± 0.9	6.2 ± 1.0	NS
PaCO_2_ kPa	8.2 ± 0.9	10.5 ± 1.6	p < 0.05
APACHE* II	16.2 ± 4.0	21.4 ± 3.0	p < 0.05
GSC**	14.0 ± 1.0	11.6 ± 1.2	p<0.05

APACHE

* Acute Physiology and Chronic Health Evaluation

GCS

*** Glasgow coma scale. NIV = Noninvasive ventilation; NS-nonsignigicant.

In patients with successful NIV treatment there is significantly higher initial GCS score, increased blood pH, lower APACHE II score and decreased PaCO_2_.

**Table 3:** Comparison of the clinical and laboratory characteristics between both groups at 1 and 4 h

Parameters	Group1 successful NIV (n=40)	Group 2 failed NIV (n=18)	P-value
After 1 hour			
Respiratory rate	23.5 ± 6.2	35.5 ± 3.0	p < 0.05
Heart rate	109.3 ± 6.9	117 ± 7.9	NS
pH	7.32 ± 0.06	7.22 ± 0.07	p < 0.05
PaO_2_ kPa	7.5 ± 0.9	7.4 ± 0.5	NS
PaCO_2_ kPa	8.0 ± 0.8	11.5 ± 1.2	p < 0.05

**Parameters**	**Group1 successful NIV (n=40)**	**Group 2 failed NIV (n=18)**	**p-value**

After 4 hours			
Respiratory rate	24.1 ± 3.2	34.8 ± 3.5	p < 0.05
Heart rate	95.9 ± 6.1	118 ± 5.4	p < 0.05
pH	7.33 ± 0.05	7.21 ± 0.04	p < 0.05
PaO_2_ kPa	7.9 ± 0.5	7.8 ± 0.4	NS
PaCO_2_ kPa	7.2 ± 0.5	10.5 ± 1.6	p < 0.05

**Figure 1:**
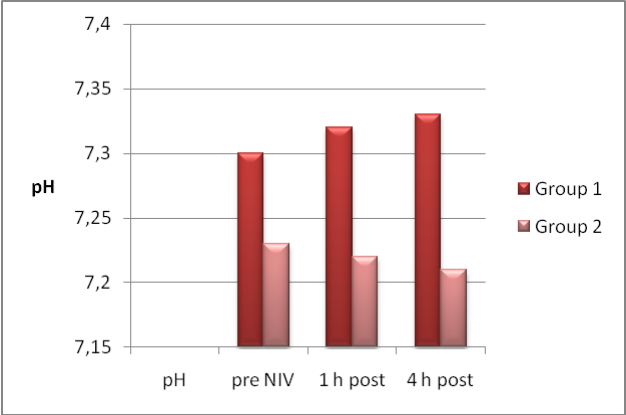
*pH pre and post NIV*.

**Figure 2:**
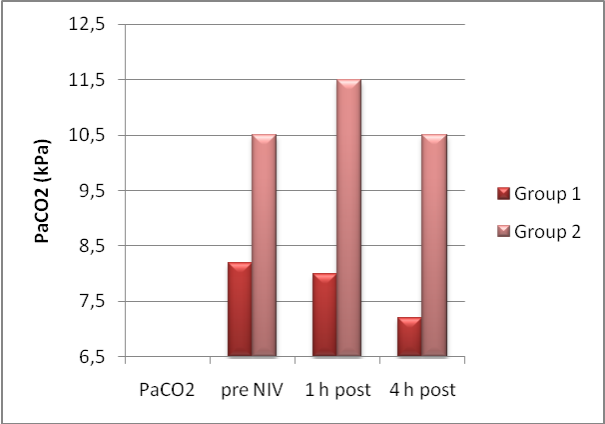
*PaCO2 pre and post NIV*.

**Figure 3:**
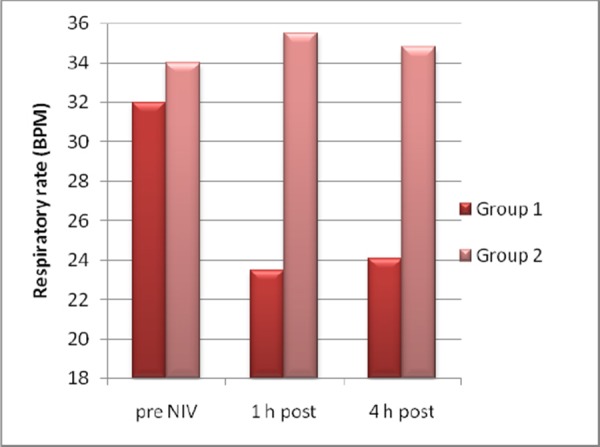
*Respiratory rate pre and post NIV*.

On admission arterial pH and Glasgow coma scale (GCS) levels were lower (pH: p<0.05, GCS: p < 0.05), and Acute Physiology and Chronic Health Evaluation II (APACHE) score and PaCO2 were higher (p < 0.05) in the NIV failure group. Arterial pH was lower (p < 0.05) and PaCO2and respiratory rate were higher (p < 0.05) after 1h, and arterial pH was lower (p < 0.05) and PaCO2 (p < 0.05), respiratory and heart rate were higher (p < 0.05) after 4h in the NIV failure group (Fig. 1, 2, 3).

Out of the results we may conclude that: GCS < 12, APACHE II > 24, pH < 7.29, PaCO_2_ >10.0 kPa at admission, as well as RR > 30/min, pH < 7.25, PaCO_2_ > 10.5 kPa, at the first hour, is in close correlation with treatment failure. During the 4 hour this pertains to RR > 30/min, pH < 7.24, PaCO_2_ >11.0 kPa, and HR >100/min.

In the group with failed NIV treatment, there was a 33.3% mortality rate, that is, in total there was a lethal outcome in six patients, while in the other group was not observed lethal outcome.

## Discussion

According to the recommendations of the Global Initiative for Chronic Obstructive Lung Disease (GOLD), the commencement of the NIV treatment is needed in every patient with decompensated COPD that fulfils the criteria for it [15]. During the follow-up if the treatment success, being aware of the predictive factors that eventually point out treatment failure, shall help in the decision-making in terms of whether the NIV treatment should be continued, that is, whether a shift in the treatment modality should be done. This is important, if we take into account that any delay of the intubation can lead to a negative outcome [27, 28].

The results of the conducted study have shown that in 68.9% of patients with decompensated COPD, a successful treatment with NIV was achieved, whereas in 18 patients there was a treatment failure (early failure in the first 48 hours of the treatment with NIV in 12 patients (20.6%) and late failure after 48 hours in six patients (10.3%)). Up to now, the conducted studies have revealed that the success rate with NIV ranges between 60-90% [9, 16-20], despite the fact that there are studies showing no effect with NIV in comparison with standard treatment [21]. The mortality rate in our study was estimated 33.3% in the group comprising the failed NIV treatment, in particular. Morreti et al., [24] in a study which had included a total of 137 patients treated with NIV because of acute exacerbation of COPD, the condition of 106 (77%) patients improved. The condition was worsened in 23% of patients, out of which, those who were switched on mechanical ventilation, a mortality rate was 53% against 92%, which is the mortality rate of patients who continued the NIV treatment.

In our study, age did not correlated with the NIV treatment effectiveness, which corresponds with the other authors’ findings [22, 23], leading to the conclusion that age should not be considered as a limiting factor for NIV treatment. We have analyzed and compared the results of both groups on admission, whereas we drew a conclusion that patients in the group with successful NIV treatment have significantly higher values of arterial pH and GCS and lower values for PaCO_2_ and APACHE II score, in comparison with the other group with failed NIV treatment. The analyzes made one hour after the NIV treatment revealed higher values for the arterial pH and lower for the respiratory rate (RR) and PaCO_2_ in the successful NIV treatment group. Four hours after the NIV treatment, the arterial pH was higher, while PaCO_2_, RR and HR were lower in the successful against the failed NIV treatment group.

According to the findings of our study, that are corresponding to the findings of many other similar studies, the starting point for patient identification that could benefit from NIV treatment should be the degree of acidosis. Furthermore, the improvement of this parameter at the first hours of the treatment, along with the decrease in respiratory rate is a predictor for a positive outcome [29]. According to Abrosino et al., [20] the initial pH has significant predictive value with sensitivity of 97% and specificity of 71%.

Antonio et al., [24] after analyzing 44 episodes of hypercapnic respiratory failure has found high correlation of the NIV treatment outcome with the PaCO_2,_ pH and the level of consciousness one hour after the ventilation. The improvement of the level of consciousness, which is most probably a consequence of the PaCO_2_ decrease one hour after the NIV treatment, has shown strong correlation as well. Identical to the study of Ambrosino et al., [20], our study findings revealed higher GCS score in patients from the successful NIV treatment group against the failed NIV group that is in accordance with the American Thoracic Society (ATS) consensus [25] stating that the altered state of consciousness should be relative contraindication for NIV.

In conclusion, in this study, being conducted for the first time in the field of NIV in our country, we aimed to present our experiences. We concluded that the measurement of certain parameters, especially pH, respiratory rate, PaCO_2_ and the state of consciousness may be of value in terms of predicting the effectiveness of NIV treatment, in order to avoid the delay the treatment with invasive mechanical ventilation, thus enabling better clinical management of the affected patients. We believe that the further studies carried out in this field and the increasing experience of clinicians will lead to a better effectiveness of the NIV treatment.
